# Juvenile Psychopathy and Community Treatment Response in Youth Adjudicated for Sexual Offenses

**DOI:** 10.1177/0306624X21994066

**Published:** 2021-02-15

**Authors:** Erika Y. Rojas, Mark E. Olver

**Affiliations:** 1 Private Practice, Saskatoon, SK, Canada; 2 University of Saskatchewan, Saskatoon, Canada

**Keywords:** Psychopathy Checklist: Youth Version, juvenile psychopathy, sexual offending, treatment, recidivism

## Abstract

The present study examined the association of juvenile psychopathy features and treatment response in a sample of 102 youth, court adjudicated for sexual offenses and followed up more than 11 years in the community. The Psychopathy Checklist: Youth Version (PCL: YV) was rated from comprehensive archival sources, along with a youth sexual offense risk assessment and treatment planning measure scored pre-and posttreatment. The PCL: YV converged with domains of sexual offense risk and change in conceptually meaningful ways, and significantly predicted nonsexual violent, general violent, and any recidivism; it did not significantly predict sexual recidivism. Higher levels of psychopathy-related personality features were significantly associated with noncompletion of youth sexual offense-specific treatment, while changes in risk were associated with decreased recidivism controlling for PCL: YV score and baseline risk at *p* < .10. The findings underscore the importance of intervention and support services for youth convicted of sexual offenses as well as the clinical and risk relevance of the juvenile psychopathy construct to decrease violent victimization to others.

The problem of sexual violence is a pressing social concern with substantial health, justice, and community safety implications. The most recent Canadian statistics available demonstrate that youth are overrepresented in the commission of sex crimes; in 2014, they accounted for 17% of persons accused of sex crimes and 26% of sex crimes against persons under age 18, most of which (>80%) were for contact offenses (Allen & Superle, 2016). In 41% of instances, the victim was a child under the age of 12 and most victims were a friend/acquaintance (64%) or family member (31%). The consequences of sexual violence are profound, with the results of meta-analyses documenting significant injurious psychological (Amado et al., 2015) and physical (Irish et al., 2010) health effects.

Juvenile psychopathy refers to a problematic pattern of interpersonal (e.g., impression management, egocentricity, lying, manipulation), affective (e.g., callousness, lack of prosocial emotions, inadequate guilt), and behavioral (e.g., risk taking, impulsivity, serious rule violations, poor anger controls) features. The traits are extreme, atypical, and maladaptive variants of the personality, emotional, and behavioral attributes characteristic of the developmental period of adolescence (Forth et al., 2003). Perhaps unsurprisingly, youth with elevated features of psychopathy are overrepresented in the youth justice system (Forth et al., 2003) and are more likely than youth with relatively few features to commit varied harms against family members, peers, or other members of the public, including sexual offenses (Caldwell et al., 2008).

The present study examines the forensic and therapeutic role and relevance of the juvenile psychopathy construct among youth with formal criminal histories of sexual offending. The following review situates the juvenile psychopathy construct, particularly as measured by the Psychopathy Checklist: Youth Version (PCL: YV; Forth et al., 2003)—a prominent symptom-construct rating scale of juvenile psychopathy designed to assess the interpersonal, affective, and behavioral features of psychopathy in male and female adolescents between ages 12 and 18—within the youth sexual offending, assessment, and intervention literatures.

## The Role and Relevance of Juvenile Psychopathy in Youth Sexual Offending

Juvenile psychopathy is a construct with forensic and clinical relevance, and it comes with a host of correlates that equate to harmful impacts including sexual as well as other forms of interpersonal violence (Edens et al., 2007; Gretton et al., 2001; Olver et al., 2009). In his review, DeLisi (2009) presents a compelling case for psychopathy as an explanatory theory for criminal behavior, given that the syndrome accounts for a disproportionate amount of varied, often instrumental, antisocial behavior across the lifespan. Juvenile psychopathy remains a robust predictor of delinquency and criminal behavior across different operationalizations of the construct (Geerlings et al., 2020), and trajectory research demonstrates an early onset of antisocial behavior that can persist into adult years (McCuish et al., 2015).

Research demonstrates that the pattern of antisociality of juvenile psychopathy can extend to sexual offending, although studies have differed in profiles of juvenile psychopathy among sexual offending and nonsexual offending youth. In a Portuguese youth justice sample, Barroso et al. (2021) found that youth convicted for sexual offenses had lower scores on the PCL: YV than youth convicted for nonsexual offenses (*M* = 14.9, *n* = 140 vs. 16.8, *n* = 130 respectively), particularly on the lifestyle and antisocial features. Youth with peer/adult victims also scored higher than youth with child victims overall and on the antisocial features, consistent with findings on profiles of psychopathy among adults convicted for sexual offenses (e.g., Olver & Wong, 2006; Porter et al., 2000). By contrast, in a Canadian sample of court adjudicated youth, Cale et al. (2015) found that youth convicted for sexual offenses had higher levels of PCL: YV-measured juvenile psychopathy than other youth (*M* = 26.0, *n* = 40 vs. 20.8, *n* = 223, respectively). Sexual offending youth were comparable to chronic violent offending youth and could be distinguished by their scores on the interpersonal and affective dimensions of the PCL: YV. It was unclear if the findings for Cale et al. (2015) may have been moderated by victim profile as they did not examine this, but the sample appeared to be more serious than youth samples elsewhere. As a point of comparison, other findings examining file rated PCL-YVs on youth sexual offending samples, both in Canada (e.g., Gretton et al., 2001, *M* = 21.2, *SD* = 7.3, *N* = 220) and the US (e.g., Viljoen et al., 2009, *M* = 17.1, *SD* = 6.1, *N* = 193), have documented scores lower than Cale et al. (2015) but higher than Barroso et al. (2021), the latter of which had a preponderance of youth with young child victims.

Pathways research such as that of Knight and Sims-Knight (2005) have demonstrated callous and unemotional features to be part of a pathway to sexually aggressive behavior in youth. McCuish et al. (2014) further identified sexual offending to be associated with different antisocial pathways, including low antisocial, overt (e.g., physical aggression), and covert (e.g., theft) pathways. The criminal trajectories of youth identified in this Canadian sample included low rate, bell shaped, slow rising chronic, and high rate chronic offending trajectories (McCuish et al., 2016); the proportion of youth identified within each trajectory who had been convicted of sexual offenses, vs. other nonsexual crimes, did not differ. Comparisons among the four trajectories by Corrado et al. (2015) further demonstrated youth in the high rate chronic trajectory to score highest on the PCL: YV and its affective, lifestyle, and antisocial domains.

## Risk Assessment Considerations of Juvenile Psychopathy for Sexual Offending Youth

The PCL: YV has generated substantial research that has implications for youth risk assessment and management. Results from meta-analysis support the predictive accuracy of PCL: YV total scores for crime and violence in forensic youth samples with moderate effects (mean weighted *r*) for general recidivism (*r_w_* = .24, Edens et al., 2007; *r_w_* = .28, Olver et al., 2009), violent recidivism (*r_w_* = .25, Edens et al., 2007; Olver et al., 2009), nonviolent recidivism (*r_w_* = .16, Olver et al., 2009), and institutional aggression (*r_w_* = .25), institutional physical violence (*r_w_* = .28), and total institutional misconducts (*r_w_* = .24) (Edens & Campbell, 2007). The existing body of research suggests that the lifestyle and antisocial features, subsumed by PCL: YV Factor 2, have greater predictive accuracy for recidivism relative to the interpersonal and affective features, subsumed by Factor 1, although a smaller volume of studies exist systematically examining this issue (Edens & Campbell, 2007; Edens et al., 2007).

Far fewer studies have examined the PCL: YV in the prediction of sexual recidivism among youth with histories of sexual offending. The Edens et al. (2007) and Olver et al. (2009) meta-analyses each examined overlapping, but slightly different, sets of studies (*k* = 4) that evaluated the predictive validity of the PCL: YV for sexual recidivism, generating *r_w_* = .07; taking into consideration the low base rates of sexual recidivism in these samples (≈13%), this translates into a *d* = .21 or area under the curve (AUC) = .56, both small effects. Of note, within these studies, PCL: YV scores had moderate to large effects for general and violent recidivism consistent with the literature on nonsexual offending youth samples. Studies conducted since have found the PCL: YV to have highly variable effects for the prediction of sexual recidivism that range from null effects (AUC = .49, Viljoen et al., 2009; AUC = .51, Cook, 2010) to large effects (*r* = .36, Caldwell et al., 2008; AUC = .77, Wijetunga et al., 2018).

Within the broader sexual offending literature, quantitative and narrative reviews have identified sexual deviance (i.e., a construct encompassing atypical sexual interests and problems with sexual self-regulation) and general antisociality (i.e., a generic risk-need domain reflecting a broad propensity for rule violation) as two core risk-need domains that underpin sexual recidivism risk (Hanson & Morten-Bourgon, 2005). By way of extension, several lines of research, primarily with adult male samples, have found PCL-R measured psychopathy and sexual deviance to have an additive or interactive effect in potentiating risk for future sexual and general violence. Hawes et al. (2013), in their meta-analysis of the psychopathy-sexual deviance nexus obtained large effects demonstrating a 2.80 to 3.21 increase in the odds of future sexual violence when both domains were present. The association is not as firmly established in the youth literature, with one study by Gretton et al. (2001) in a sample of 220 youth convicted for sexual offenses, who found that PCL: YV score and phallometric indices of sexual deviance had additive effects in the prediction of general and violent recidivism, but not sexual recidivism.

Finally, a growing literature has identified a pattern for youth with substantial psychopathic features to be at increased risk for sexually victimizing their peers within institutional settings. A US-based dissertation (Farr, 2013) examining institutional sexual misconduct in a sample of 389 youth convicted for sexual offending attending a residential treatment program found that youth who committed sexual misconducts had significantly higher PCL: YV scores in general (*d* = .41) and on the interpersonal facet in particular (*d* = .59). The study contributes to the broader literature demonstrating that in addition to posing a risk to the community and public safety, that youth with elevated psychopathic traits can also pose a risk for sexual harm directed toward staff and peers within institutional settings. What is the solution? It should logically follow that this would be treatment and risk management.

## Treatment Responses of High Psychopathy Youth

The construct of juvenile psychopathy has understandably been the focus of some controversy in clinical, legal, and academic circles. Among expressed concerns are that some variants of the traits are potentially normative (e.g., egocentricity, risk taking) (Salekin, 2006; Seagrave & Grisso, 2002; Vincent & Hart, 2002), that adolescence is a period of massive developmental change (Edens & Vincent, 2008; Hart et al., 2002; Vincent & Hart, 2002), and issues of diagnostic comorbidity that make it difficult to disentangle the symptoms of other mental disorders from psychopathic features (Salekin, 2006; Salekin & Frick, 2005; Seagrave & Grisso, 2002; Vincent & Hart, 2002). Most importantly, perhaps, labelling youth “psychopathic” can have detrimental clinical and legal consequences such as transfer from juvenile to adult court, harsher sentences, negative perceptions of treatment amenability, and limited accessibility to treatment (e.g., Murrie et al., 2005; Penney & Moretti, 2005; Rockett et al., 2007; Seagrave & Grisso, 2002; Skeem & Cauffman, 2003; Viljoen et al., 2010; Vincent & Hart, 2002; Vitacco & Vincent, 2006). That said, there are divergent perspectives, given that the traits of juvenile psychopathy are extreme and maladaptive variants of “normal” personality (Forth et al., 2003), juvenile psychopathy has myriad correctional and criminal justice correlates (Edens et al., 2007; Geerlings et al., 2020; Olver et al., 2009), and some lines of judicial and mock juror research have found no support for possible prejudicial effects of the “psychopathy” label for youth or adults (e.g., Cox et al., 2010; Fairfax-Columbo, 2017; Murrie et al., 2007).

Therapeutic pessimism abounds regarding the treatment of high psychopathy individuals, and much of the available literature has featured adult male samples (Olver, 2016; Salekin, 2002; Salekin et al., 2010). The available literature has demonstrated that this group tends to be a particularly challenging population to treat and are more likely to display low motivation (Ogloff et al., 1990), make fewer treatment gains (Olver et al., 2013), and have higher rates of attrition (Olver et al., 2011). In treated adult male sexual offending populations, scores on the affective features of psychopathy have been associated with increased program attrition (Olver & Wong, 2011), decreased therapeutic progress (Sewall & Olver, 2019), and weaker working alliances, particularly the emotional bond between client and therapist (DeSorcy et al., 2020). Importantly, however, when high psychopathy clientele can be retained in correctional treatment programs that follow established practices, such as the risk, need, responsivity (or RNR) principles, program related gains have been associated with decreased recidivism (Langton et al., 2006; Looman et al., 2005; Olver et al., 2013; Skeem et al., 2002; Wong et al.,2012).

Meta-analyses of the adolescent sexual offending treatment literature (Reitzel & Carbonell, 2006), the general juvenile offending treatment literature (Lipsey, 2009), and high intensity individualized treatment regimes such as Multisystemic Therapy (MST; van der Stouwe et al., 2014) have documented therapeutic effects in terms of recidivism reduction. Less research has examined psychological treatment of justice involved youth as a function of psychopathy, however. In a sample of 253 youth attending a residential sexual offense treatment program followed up for 10 years in the community post program, Gretton et al. (2005) found that treatment completers had lower rates of sexual recidivism than noncompleters (14% vs. 27%, respectively). Although youth with elevated psychopathic traits were significantly less likely to complete treatment, those youth who were retained in services had lower rates of subsequent violent reoffending than similarly high psychopathy youth who did not complete services (33% vs. 83%, respectively). Further, the work by Caldwell et al. (2006, 2007) with youth convicted for violent offenses attending a high intensity residential program, found that a subgroup of youth with many psychopathy-related personality features showed a positive response to appropriate treatment, specifically, improved treatment compliance and reduced aggression. Taken together, these findings suggest that early intervention may be beneficial for youth, and that contrary to the historically pessimistic viewpoint, youth with substantive psychopathy features can respond favorably to treatment services to reduce societal victimization and harmful impacts on others.

## Present Study

Few studies have examined the forensic and therapeutic role and relevance of the juvenile psychopathy construct among court adjudicated sexually offending youth. The present study examined exactly this—treatment response and prediction of recidivism in a sample of youth referred for sexual offense services as a function of PCL: YV-measured psychopathy followed up approximately 11 years in the community. The study further examines the sexual violence risk-need correlates of juvenile psychopathy and the association of treatment completion and treatment-related changes in risk with program outcomes. We argue that juvenile psychopathy, operationalized via the PCL: YV, can inform risk assessment, intervention, and management among youth with histories of sexual offending to increase prosocial functioning and wellbeing, and to decrease violent victimization and harmful effects to others. Specifically, high PCL: YV scoring youth would be candidates for more intensive services (risk principle), have a greater number of criminogenic needs to inform the content and foci of services (need principles), and present with interpersonal and affective features that necessitate flexible and skillful adaptation of services to minimize attrition and maximize potential for benefit (responsivity principle).

## Method

The present research involved a multistep process that entailed obtaining approvals from a Saskatchewan Provincial Court Judge, the local health region of the Saskatchewan Health Authority, and ethical approval from the University of Saskatchewan’s Behavioural Research Ethics Board (BEH# 10-200).

### Participants

Participants were 102 male youth, based on consecutive admissions, that had received outpatient services (i.e., assessment and/or treatment) between 1995 and 2008 from a young offender program at a community mental health facility through the Saskatchewan Health Authority. Two cases were excluded owing to substantial missing data (including PCL: YV score) bringing the sample to *N* = 100. The mean age at the time of service was 16.24 years (*SD* = 1.97, range 12–19), and 14.30 years (*SD* = 1.90) at the time of the index sexual offense that prompted referral. All youth had a court adjudicated conviction for a sexual offense as their index offense (e.g., sexual assault with a weapon, sexual assault, sexual interference, invitation to sexual touching, incest, indecent exposure), or in few instances, a sexually motivated offense adjudicated as a nonsexual offense (i.e., assault). Most youth had a conviction for sexual assault (*n* = 70, 70%), followed by sexual interference (i.e., touching a body region for sexual purposes, *n* = 20, 20%), sexual assault with a weapon (*n* = 6, 6%), and the remainder convicted for other categories of sexual offenses and/or sexually motivated offenses pled down to nonsexual offenses (*n* = 4, 4%). Youth had an average of 2.10 index convictions (*SD* = 3.58) and 80% had the lone sexual offense(s) as their index offense(s). Youth had an average index sentence length of 24.19 months (*SD* = 5.84, *n* = 81) and were most frequently sentenced to probation (60.6%). The ethnocultural composition of the sample was Caucasian (43%), Indigenous (23%), and 34% of unknown descent.

### Adolescent Sexual Offense Treatment Program

About two-thirds of the sample (*n* = 61, 61%) attended therapeutic sexual offense services and only five youth received treatment in other settings elsewhere in the province (e.g., closed custody, residential treatment program). Fifty five (55%) and 52 (52%) youths participated in individual and group treatment, respectively, targeting sexual offending behavior following assessment. Regarding sexual offense group treatment, youth frequently participated in a psychoeducational group followed by a relapse prevention group (number of sessions ranged from 10 to 12 per group) if the former group was successfully completed. Group treatment followed a cognitive-behavioral treatment (CBT) framework. Program components included cognitive restructuring, victim empathy, communication skills including assertiveness, anger management, development of an individualized offending cycle and relapse prevention plan. Of note, interventions from the treatment program were risk informed, that is, treatment issues were identified for each youth based on the available knowledge and assessment technology at the time. Individual services were offered frequently and were tailored to client-specific issues.

Youth attended treatment services during and until their probation expired (typically a 2-year period). When their probation expired, then treatment services were completed. If youths attended the psychoeducational/cognitive behavioral group and the relapse prevention group, and/or attended individual sessions before, between, or following the groups, then they completed full services. Youth were initially coded as successfully completing none, partial, or full treatment services. Decisions pertaining to successful completion of treatment were based primarily on treatment providers’ assessment of youth’s participation in one or more modes of sexual offense treatment (group and/or individual). For instance, partial treatment services could entail a successful completion of the psychoeducational group, but not the relapse prevention group. Youth who successfully completed none or partial services were combined to form a noncompletion group; of note, youth considered to have successfully completed “none” of the treatment services and received group services typically attended only a few group sessions. Reasons for not completing full services included missing group sessions, poor participation, minimal engagement, and/or disruptive behavior.

#### Psychopathy Checklist: Youth Version (PCL: YV)

The PCL: YV (Forth et al., 2003) is a 20-item rating scale designed to assess psychopathy-related personality features in youth between the ages of 12 and 18. Items measure the interpersonal, affective, and behavioral features of the psychopathy construct. Each item is rated on a 3-point scoring system: 0 (“definitely does not apply”), 1 (“applies to some extent”), and 2 (“definitely applies”). Scores for individual items are summed to obtain a total score ranging 0 to 40. Although the PCL: YV provides dimensional scores, Forth et al. (2003) note that youth can be categorized based on the degree of psychopathic traits present for research purposes. For instance, Gretton et al. (2001) divided youth into low (<18), medium (18–29), and high (≥30) PCL: YV groups based on their total scores. The PCL: YV Total and Factor scores can be examined separately. Research shows that the three- and four-factor models are good representations of the internal structure of the PCL: YV (Jones et al., 2006; Kosson et al., 2013; Neumann et al., 2006; Sevecke et al., 2009). It is recommended to use file and interview data to rate the PCL: YV, particularly for the interpersonal features for clinical use. Nevertheless, a well-established body of research supports the validity and reliability of file-rated PCL: YV scores from sufficiently detailed archival information sources (e.g., Catchpole & Gretton, 2003; Gretton et al., 2001, 2005). Fair to good interrater reliability (<.40 poor, .40–.59 fair, .60–.74 good, and .75–1.0 excellent; Cicchetti & Sparrow, 1981) was obtained for PCL-YV total scores on 16 randomly selected double coded cases: Intraclass correlation, two-way random effects, single measure, absolute agreement (ICC_A1_) = .59, and consistency agreement (ICC_C1_) = .65.

#### Violence Risk Scale: Youth Sexual Offense Version (VRS-YSO)

The VRS-YSO (Olver et al., 2017) is a 23-item sexual offense risk assessment and treatment planning tool for youth adjudicated for sexual offending. The VRS-YSO contains 6 static and 17 dynamic items. Each risk item was rated on a 4-point ordinal scale (ranging from 0 to 3), where higher ratings indicated greater prevalence of risk factors associated with an increased risk for sexual offending. Dynamic items receiving a 2 or 3 rating are considered criminogenic and to be prioritized for treatment, while 0 or 1 ratings reflect a low risk item not prioritized for services. Identified treatment targets receive a baseline Stage of Change rating (Prochaska et al., 1992) that reflects the youth’s motivation and readiness to change according to five stages: precontemplation (denial of problem area, lack of insight), contemplation (awareness of problem area), preparation (recent or inconsistent use of skills and strategies to manage the problem area), action (sustained use of skills and strategies), or maintenance (generalization and transfer of learning across different contexts). The Stage of Change is then re-rated at posttreatment for the same identified treatment targets. Progression from one stage to the next stage, reflecting improvement, is credited with a 0.5-point decrease in risk rating, 1.0 points for two stages and so on; regression from one stage to the next in the direction of deterioration is associated with a corresponding increase in risk score. A change score can be obtained by summing the change ratings across the 19 dynamic items.

Research examining the structural and predictive properties of the VRS-YSO from this sample (Rojas & Olver, 2020) found that risk scores predicted sexual, violent, and general recidivism (AUCs = .65–.79) and that the items could be arranged into three oblique factors termed: Sexual Deviance, Antisocial Tendencies, and Family Concerns. Good interrater reliability, per Cicchetti and Sparrow (1981), was obtained from randomly selected double coded cases (*n* = 16 for static and pre, *n* = 11 for post) as follows: Static ICC_A1_ = .71, Dynamic (pre) ICC_A1_ = .65, (post) ICC_A1_ = .69, Total (pre) ICC_A1_ = .74, (post) ICC_A1_ = .72.

#### Recidivism variables

Recidivism was coded from the youths’ criminal records obtained through the Canadian Police Information Centre (CPIC), Canada’s nationwide database of criminal charges and convictions maintained by the Royal Canadian Mounted Police (RCMP). Recidivism was defined as any court adjudicated criminal code conviction incurred following the youths’ commencement of services at the agency (for community sentenced youth) or post-release (for youth with custodial sentences). Sexual recidivism was defined as any new sexually motivated offense (e.g., sexual assault, sexual interference) including noncontact sexual offenses. Nonsexual violent recidivism consisted of any new offense with the potential for physical or psychological harm that was nonsexually motivated (e.g., robbery, nonsexual assault). General violent recidivism was defined as any new sexual or nonsexual violent offense. Finally, any recidivism consisted of a new offense from any category (i.e., sexual, nonsexually violent, or nonviolent). The use of convictions vs. charges (i.e., the youth is arraigned on a criminal code matter but is not determined to be guilty either through plea or trial for various reasons), did not impact sexual recidivism base rates in the sample (i.e., all new sexual offense charges subsequently resulted in conviction and sentencing) or other outcomes.

Although CPIC has its advantages with the depth and comprehensiveness of its coverage, it can underestimate rates of youth recidivism given that youth records are sealed either after 3 years (for a summary conviction) or 5 years (for an indictable offense) of inactivity unless new criminal code charges or convictions are incurred as an adult within the time window (Department of Justice Canada, 2017). As the youth CPIC records were updated and obtained more than a decade on average following the youth’s initial assessment, a given youth may not have an active record on file either because they did not reoffend or due to the record being sealed (i.e., even if they did reoffend as youth, but did not offend as adults). The trade-off is an underestimate of youth recidivism that is partly offset by more accurate estimates of long-term (i.e., adult) recidivism.

The number of months youth were on probation and/or free in the community was recorded to calculate the total number of months they spent in the community during follow-up. The date of new recidivistic offenses was coded for a given offense category to calculate time in the community to next offense in order to conduct survival analysis. The time duration for non-recidivists for a given recidivism category was the time duration from the first exposure to risk post assessment to the CPIC data capture date.

#### Victim age

Finally, the sample was classified according to victim age (based on history and index sexual offenses) into youth who offended exclusively against children vs. those who offended against peers or adult, in light of differences often found in profiles of psychopathy in the adult sexual offending literature (e.g., Olver & Wong, 2006; Porter et al., 2000). Based on Prentky and Righthand’s (2003) criteria, a young child victim was ≤10-years-old and ≥4 years younger than the abuser at the time of the offense, while a peer or adult victim was ≥11 years-old at the time of the offence.

### Procedure

The sample represented consecutive admissions for outpatient sexual offense assessment and/or treatment services between 1995 and 2008. The youths’ court and treatment files, which were opened and generated for the purpose of these services, were carefully reviewed to retrospectively code the PCL: YV, VRS-YSO, participant characteristics, and treatment information. Ratings of all study measures were completed by the first author (who was a clinical psychology graduate student at the time of the research) and a senior undergraduate psychology student, both of whom were trained in rating the study measures by the second author, a registered psychologist with several years experience working clinically with court adjudicated youth. The youth files included psychological assessments, school records, social history reports, treatment progress notes, and court and legal documents (e.g., presentence reports). File quality was rated on a random sample (*n* = 56) using a 5-point Likert scale (1 = very poor quality to 5 = very good quality). File quality was good in general (*M* = 4.06, *SD* = 1.11, Mode = 5), although 12.5% (*n* = 7) were rated poor (i.e., score 2) and 3.5% (*n* = 2), very poor (i.e., score 1); no assessment measures could be coded from very poor files. Coding of all measures was completed prior obtaining criminal recidivism data, and thus, both raters were blind to outcome.

### Planned Analyses

First, we conducted group comparisons on PCL: YV facet and total scores as a function of victim profile (i.e., peer vs. adult) through *t*-test and standardized mean difference (Cohen’s *d*) comparisons. We also drew comparisons between the PCL: YV profiles in the current sample to that of other youth sexual offending samples. Second, we examined the risk and need correlates of latent psychopathic traits through computing bivariate correlations between PCL: YV facet and total scores with the VRS-YSO factor, static, dynamic, and total scores. Positive correlations would indicate measurement of a common construct and hence, shared risk variance. Using Cohen’s (1992) conventions, correlations of .10, .30, and .50 correspond to small, medium, and large effect sizes, respectively.

Third, we examined the predictive accuracy of PCL: YV facet and total scores for sexual, nonsexual violent, general violent, and any recidivism through receiver operating characteristic (ROC) analyses. ROC generates an area under the curve (AUC) statistic ranging from 0 to 1.0 that represents the probability a randomly selected recidivist would have a more deviant score on an assessment measure than a randomly selected non-recidivist. With an AUC of .50 representing chance level predictive accuracy, values of .56, .64, and .71 correspond to small, medium, and large effect sizes, respectively (Rice & Harris, 2005).

Fourth, we extended the predictive validity analyses to examine the extent to which PCL: YV scores may increment the prediction of the four recidivism outcomes beyond an actuarial measure developed for the purpose of assessing risk for sexual recidivism, such as the VRS-YSO. Cox regression survival analysis was conducted in which VRS-YSO total score (i.e., static + dynamic) was entered in the first block followed by the PCL: YV total score in the second block; the order of entry for the predictors was then reversed to ascertain the main effect for PCL: YV score absent other covariates. Given that only a little more than half of the sample had posttreatment information available, VRS-YSO pretreatment scores were employed to make use of the full sample and to maximize power. Cox regression generates a hazard ratio (*e*^*B*^), representing the proportionate change in hazard for every one-point increase in the predictor; values above 1.0 indicate a positive association between the predictor and criterion and values below 1.0 indicate an inverse association. If the PCL: YV increments the prediction of recidivism, evidence would be that the total score significantly predicts a given outcome after controlling for the actuarial risk measure.

Fifth, we investigated the therapeutic correlates of PCL: YV scores, through examining bivariate and multivariate associations between PCL: YV facet scores with: (i) sexual offense treatment program completion and (ii) treatment related changes as measured by VRS-YV pre-post change scores. As the adult psychopathy treatment literature has demonstrated PCL-measured psychopathy to be associated with increased risk for noncompletion and deceased therapeutic progress, the present study examined to what extent these associations were present in a youth treatment sample.

Finally, we conducted a set of Cox regression survival analyses to examine the associations of—(i) treatment completion and (ii) treatment-related change—to recidivism controlling for baseline risk (VRS-YSO total score) and psychopathy (PCL: YV total score). In light of the small *n* for these analyses (e.g., *n* = 53 with VRS-YSO change scores) which compounds the base rate and power issue, particularly for multivariate analyses, we examined general violent and any recidivism only (i.e., the two highest base rate outcomes). For the first set of Cox regressions, youth scoring above average in the sample on the PCL: YV total who did not complete full services were used as a reference group for comparison to above average PCL: YV youth who did complete full services, as well as the remaining lower scoring youth. Indicator contrasts were used to compare survival trajectories and VRS-YSO total was entered as a continuous covariate to control for baseline risk. The second set of Cox regressions entered PCL: YV total score, VRS-YSO pretreatment total (i.e., static and dynamic), and pre-post change score as covariates in the prediction of the two recidivism outcomes over time. These latter analyses would examine to what extent changes in risk, measured pre-posttreatment, would be associated with possible reductions in recidivism after controlling for baseline risk and individual levels of juvenile psychopathy. If changes in risk are associated with decreased recidivism irrespective of psychopathy, then PCL: YV and VRS-YSO should be uniquely positively associated with recidivism, while pre-post risk change scores should be uniquely negatively associated with outcome.

## Results

### Sample Profile and Description

Table 1 reports the means and standard deviations for the PCL: YV for this youth sample as a whole and as a function of victim profile. The sample had lower PCL: YV total scores on average (*M* = 12.9, *SD* = 6.5) compared to other juvenile sexual offending samples both in Canada (e.g., Cale et al., 2015; Gretton et al., 2001) and the US (e.g., Viljoen et al., 2009), although the profiles were similar to Barroso et al.’s (2021) Portuguese sample. This may be attributable to the sample composition, given that most youth had committed sexual offenses exclusively against child victims (per Barroso et al., 2021), and scored substantially lower on the PCL: YV overall (*d* = 0.72) and on all but the interpersonal facet, compared to youth with peer age or adult victims. The latter group, by contrast, had scores that were closer in magnitude to those seen elsewhere. By and large this was a lower psychopathy sample, but with considerable variability (i.e., PCL: YV range 2–28).

**Table 1.** Descriptive Statistics for PCL: YV.

PCL: YV	Aggregate sample (*N* = 100)		Young child victims (*n* = 70)		Peer/adult victims (*n* = 26)		
	*M* (*SD*)	Range	*M (SD)*	Range	*M* (*SD*)	Range	*d*
Interpersonal	1.6 (1.2)	0–6	1.7 (1.1)	0–6	1.5 (1.1)	0–4	.18
Affective	2.8 (2.0)	0–7	2.4 (1.7)	0–7	3.9 (2.0)	0–7	.84
Lifestyle	3.7 (2.3)	0–10	3.2 (2.2)	0–8	4.7 (1.9)	1–9	.71
Antisocial	3.3 (2.2)	0–9	3.0 (2.0)	0–9	4.0 (2.4)	1–9	.47
Total	12.9 (6.5)	2–28	11.8 (6.1)	2–28	15.4 (6.3)	5–25	.72

*Note.* Youth with peer/adult victims significantly higher scores than youth with young child victims on all PCL: YV scale components except for Interpersonal facet.

### Convergent Associations between Juvenile Psychopathy and Sexual Offense Risk

Table 2 is a convergent validity correlation matrix examining associations between the PCL: YV and actuarial sexual offense risk as measured by the VRS-YSO. In short, PCL: YV scores demonstrated significant moderate to large positive correlations with the VRS-YSO static, dynamic, and total scores; somewhat larger in magnitude associations were found for the lifestyle and antisocial facets with risk scores compared to the interpersonal and affective facets. Of the three VRS-YSO factors, the strongest and most consistent associations were observed with respect to the antisocial tendencies facet; by contrast, only the interpersonal facet correlated significantly with sexual deviance, while the lifestyle and antisocial facets correlated significantly (small to moderate associations) with the VRS-YSO family concerns factor. In short, high psychopathy youth tended to be higher risk actuarially for sexual violence, but much of this association could be accounted for by scores on the general antisocial tendencies factor.

**Table 2.** Convergent Validity Correlation Matrix: Correlations between VRS-YSO Scale Component Scores and PCL: YV.

	VRS-YSO					
PCL: YV	Static	Dynamic	Sexual deviance	Antisocial tendencies	Family concerns	Total
Interpersonal	.38***	.51***	.45***	.43***	.08	.52***
Affective	.38***	.41***	.07	.59***	.04	.44***
Lifestyle	.39***	.61***	.05	.76***	.30**	.60***
Antisocial	.50***	.60***	.14	.76***	.22*	.62***
Total	.56***	.69***	.21	.83***	.23*	.71***

*Note.* VRS-YSO scores represent pretreatment rated measures; *n*s = 96 and 98 for facet and total scores, respectively.

NS = not significant.

***p ≤ .001. **p < .01. *p < .05.

### PCL: YV Predictive Accuracy for Recidivism

The sample was followed up a mean 11.7 years (*SD* = 3.4) in the community, during which the base rates of reoffending were 8% (*n* = 8) for sexual recidivism, 18% (*n* = 18) for nonsexual violent recidivism, 25% (*n* = 25) for general violent recidivism, and 39% (*n* = 39) for any recidivism. Table 3 reports predictive associations between PCL: YV score and the three recidivism outcomes through ROC analyses. None of the PCL: YV associations with sexual recidivism were significant, partly owing to decreased power given that the antisocial facet had a moderate effect size (AUC = .65) for this outcome. PCL: YV total, lifestyle, and antisocial facet scores had significant and moderate to high predictive accuracy for nonsexual violent, general violent, and any recidivism (AUCs = .66–.74); affective facet scores also significantly predicted any recidivism with a lower range moderate effect. The interpersonal facet had weak nonsignificant effects for all four recidivism outcomes.

**Table 3.** Predictive Accuracy of Risk and Change Scores on Juvenile Sexual Offense Risk Measures for Recidivism Outcomes.

PCL: YV	Sexual recidivism		Nonsexual violent recidivism		General violent recidivism		Any recidivism	
	AUC	95%CI	AUC	95%CI	AUC	95%CI	AUC	95%CI
Interpersonal	.45	.25, .66	.57	.41, .72	.54	.40, .68	.53	.41, .65
Affective	.60	.42, .78	.61	.46, .76	.62	.49, .75	.64*	.52, .75
Lifestyle	.58	.42, .75	.74**	.62, .85	.73***	.62, .83	.70***	.60, .81
Antisocial	.65	.48, .81	.70**	.57, .83	.71**	.60, .83	.66**	.55, .78
Total	.61	.42, .80	.68*	.54, .82	.69**	.56, .81	.67**	.55, .78

*Note. N* = 98.

***p ≤ .001. **p < .01. *p < .05.

A set of post hoc analyses examined the rates of recidivism associated with PCL: YV scores divided into groups based on the distribution of low (0–9, *n* = 35), average (10–19, *n* = 44), and above average (20–40, *n* = 31) scores. There were no significant group differences in rates of sexual recidivism (low 5.7%, average 6.8%, above average 14.3%; χ^2^ [2, *N* = 100] = 1.46, *ns*, AUC = .60, 95%CI = .39–.82), but youth in the above average PCL: YV group had significantly higher rates of general violent (low 14.3%, average 20.5%, above average 47.6%; χ^2^ [2, *N* = 100] = 8.45, *p* = .014, AUC = .66, 95%CI = .53–.79) and any (low 25.7%, average 34.1%, above average 61.9%; χ^2^ [2, *N* = 100] = 7.66, *p* = .022, AUC = .64, 95%CI = .53–.75) recidivism.

Finally, Table 4 reports the incremental associations of the PCL: YV and the VRS-YSO pretreatment total score to the four recidivism outcomes. Collinearity diagnostics ruled out the potential for multicollinearity given that the measures were not too highly correlated (i.e., <.85, *r* = .71), tolerance was above .10 (i.e., .50), and variance inflation factor was low (i.e., <10; VIF = 2.00) (Midi et al., 2010; Schroeder, 1990). VRS-YSO total score significantly predicted each recidivism outcome when entered in the first block (Block 1a), while PCL: YV total score significantly predicted all but sexual recidivism when entered in the first block (Block 1b). Neither measure uniquely predicted any of the outcomes when added to the second block, however, despite the increase in some effect sizes (i.e., VRS-YSO with sexual recidivism), owing to limited power.

**Table 4.** Cox Regression Survival Analyses: Incremental Associations of Juvenile Psychopathy and Actuarial Sexual Offense Risk to Recidivism Outcomes.

Regression model and outcome	*B*	*SE*	Wald	*p*-Value	*e* ^*B*^	95%CI	
Sexual recidivism							
Block 1a							
VRS-YSO pretreatment total	.08	.04	4.00	**.046**	1.09	1.002	1.18
Block 1b							
PCL: YV total	.07	.05	1.60	.206	1.07	0.96	1.19
Block 2							
VRS-YSO pretreatment total	.09	.06	2.66	.103	1.10	0.98	1.23
PCL: YV total	–.03	.08	0.10	.755	0.98	0.84	1.14
Nonsexual violent recidivism							
Block 1a							
VRS-YSO pretreatment total	.10	.04	6.75	**.009**	1.10	1.02	1.18
Block 1b							
PCL: YV total	.10	.04	7.45	**.006**	1.10	1.03	1.19
Block 2							
VRS-YSO pretreatment total	.06	.05	1.26	.262	1.06	0.96	1.17
PCL: YV total	.06	.05	1.15	.283	1.06	0.96	1.17
General violent recidivism							
Block 1a							
VRS-YSO pretreatment total	.08	.02	10.11	**.001**	1.08	1.03	1.13
Block 1b							
PCL: YV total	.10	.03	9.57	**.002**	1.10	1.04	1.17
Block 2							
VRS-YSO pretreatment total	.05	.04	2.31	.128	1.05	0.99	1.13
PCL: YV total	.05	.05	1.00	.317	1.05	0.96	1.15
Any recidivism							
Block 1a							
VRS-YSO pretreatment total	.07	.02	11.28	**.001**	1.07	1.03	1.11
Block 1b							
PCL: YV total	.09	.03	11.35	**.001**	1.09	1.04	1.14
Block 2							
VRS-YSO pretreatment total	.04	.03	2.31	.128	1.04	0.99	1.10
PCL: YV total	.05	.04	1.56	.212	1.05	0.98	1.12

*Note. N* = 98, significant *p*-values in bold font. Block 1a is entry of VRS-YSO in first step; Block 1b is entry of PCL: YV in first step; Block 2 is the final predictor model.

### Treatment Completion and Change Associations with Outcome

Table 5 reports bivariate and multivariate associations between the PCL: YV facets and VRS-YSO treatment-related risk change (*N* = 53) and treatment noncompletion (*N* = 62). As previously reported in (Rojas & Olver, 2020), the sample changed by nearly a full standard deviation (*d* = .94, *M* = 6.8, *SD* = 4.5) in their VRS-YSO score from pre to posttreatment. PCL: YV total scores were not significantly associated with VRS-YSO change scores (*r* = .03), nor were any of the facets; thus, the youths’ levels of psychopathy on the measure as a whole or the individual trait dimensions were largely unrelated to the amount of risk-related treatment change. Collinearity diagnostics ruled out the potential for multicollinearity among the PCL: YV facets as predictor variables (*r*s = .32–.60; tolerance = .49–.72; VIFs = 1.40–2.03). None of the facets significantly incrementally predicted treatment change, controlling for the other facets; however, the interpersonal facet had a moderate in magnitude positive association (*β* = .32) with change, while the antisocial facet had similar in magnitude negative association (*β* = −.27) with change.

**Table 5.** Correlation and Regression Analyses: Associations between PCL-YV Facets, Treatment Completion, and Change.

PCL-YV facet	Univariate *r*/*r*_*pb*_	*B*	*SE*	*β/e* ^B^	*t/χ* ^2^	*p*-Value
Treatment change						
Interpersonal	.17	1.42	.80	.32	1.78	.082
Affective	.04	0.15	.44	.06	0.34	.739
Lifestyle	–.06	–0.09	.36	–.04	–0.25	.805
Antisocial	.06	–0.53	.45	–.27	–1.19	.239
Treatment noncompletion						
Interpersonal	.32*	.47	.33	1.60	2.07	.150
Affective	.19	–.05	.20	0.96	0.05	.821
Lifestyle	.38**	.35	.17	1.42	4.07	**.044**
Antisocial	.27*	.18	.18	0.96	0.06	.807

*Note.* *p < .05. **p < .01. for zero order (*r*) and point biserial (*r_pb_*) univariate correlations with treatment change and noncompletion, respectively. Significant *p*-values for regressions in bold font. *β/eB* and *t/χ*^2^: standardized regression coefficient (*β*) and *t*-test (*t*) for treatment change predictors; odds ratio (*e*^B^) and Wald’s chi square (*χ*^2^) for binary treatment noncompletion predictors; *n* = 53 for treatment change analyses, *n* = 62 for treatment noncompletion analyses.

Most of the youths in the sample (*n* = 36/62, 58.1%) completed full services (i.e., both groups), while equal portions successfully completed partial services (i.e., 1 group, *n* = 13/62, 21.0%), or no services (i.e., neither group, *n* = 13/62, 21.0%). Given the modest *N*, and the fact that there was no difference in treatment change for youth who completed one (*M* = 4.1, *SD* = 3.9) vs. no groups (*M* = 4.3, *SD* = 4.2) in contrast to those who completed full services (*M* = 8.3, *SD* = 4.2) (*F* [2, 52] = 6.22, *p* = .004), a binary treatment noncompletion variable (yes-no completed full services) was utilized to examine its association with psychopathy and recidivism outcome. PCL: YV total score was significantly associated with binary treatment noncompletion (*r* = .35, *p* = .005), as were scores on the interpersonal, lifestyle, and antisocial facets. As with the multivariate change analyses, results of binary logistic regression demonstrated that interpersonal and lifestyle facet scores had meaningful associations with treatment noncompletion; specifically, the odds ratio (*e*^*B*^) was significant only for the lifestyle facet, although larger for the interpersonal facet owing to the higher standard error.

Table 6 reports the final set of analyses which examined the associations of sexual offense treatment completion and pre-post risk change to the two highest base rate outcomes (general violent and any recidivism) controlling for individual differences in levels of juvenile psychopathy and baseline risk. Although psychopathy was associated with treatment noncompletion, there were no significant differences between completers and noncompleters in rates of general violent or any recidivism as a function of psychopathy, after controlling for baseline risk (i.e., VRS-YSO total score). In the psychopathy, risk, and treatment change analyses, none of the covariates significantly incrementally predicted general violent or any recidivism. Collinearity diagnostics ruled out the potential for multicollinearity among the predictor variables (*r*s = .03–.75; tolerance = .34–.87; VIFs = 1.16–2.91). For any recidivism, meaningful findings emerged for PCL: YV total score (*e*^*B*^ = 1.11, *p* = .089) and VRS-YSO change (*e*^*B*^ = 0.92, *p* = .093) score associations with this outcome, as the hazard ratio magnitudes were consistent for incremental associations of parallel measures of these tools with outcome in larger treated adult samples (e.g., Olver et al., 2013; Sewall & Olver, 2019).

**Table 6.** Cox Regression Survival Analysis: Associations of Juvenile Psychopathy, Treatment Change, and Treatment Completion, to General Violent and Any Recidivism Controlling for VRS-YSO Pretreatment Score.

Regression model and outcome	*B*	*SE*	Wald	*p*-Value	*e* ^*B*^	95%CI	
Psychopathy and treatment completion							
General violent recidivism							
PCL: YV ≤15, Completer	–.67	.88	0.59	.510	0.51	.09	2.87
PCL: YV ≤15, Noncompleter	–.53	1.02	0.27	.602	0.59	.08	4.33
PCL: YV >15, Completer	.80	.70	1.31	.252	2.23	.57	8.83
VRS-YSO pretreatment total	.04	.04	0.89	.346	1.04	.96	1.13
Any recidivism							
PCL: YV ≤15, Completer	–.93	.73	1.62	.203	0.40	.09	1.65
PCL: YV ≤15, Noncompleter	–.59	.82	0.51	.476	0.56	.11	2.78
PCL: YV >15, Completer	.02	.62	0.00	.977	1.02	.30	3.42
VRS-YSO pretreatment total	.05	.04	2.01	.156	1.06	.98	1.14
Psychopathy and treatment change							
General violent recidivism							
PCL: YV total	.09	.07	1.54	.214	1.09	.95	1.26
VRS-YSO pretreatment total	.02	.05	0.10	.752	1.02	.92	1.12
VRS-YSO change	–.06	.07	0.95	.329	0.94	.83	1.07
Any recidivism							
PCL: YV total	.11	.06	2.90	.089	1.11	.98	1.26
VRS-YSO pretreatment total	.04	.04	0.77	.379	1.04	.96	1.12
VRS-YSO change	–.09	.05	2.83	.093	0.92	.82	1.02

*Note.* Psychopathy and treatment completion analyses *n* = 61; Psychopathy and treatment change analyses, *n* = 53. For psychopathy, treatment completion, and recidivism analyses at the top of the table, PCL: YV >15, Noncompleter group used as the reference group for indicator contrasts.

## Discussion

The present study examined the role of PCL: YV measured juvenile psychopathy in the criminal and treatment outcomes of youth who have sexually offended. From a tertiary prevention standpoint, the sample represents a group of youth who committed contact sexual offenses, most frequently against a child family member, who were referred for assessment and intervention services with the goals of: (a) preventing future victims, primarily from sexual offending; and (b) preventing the youth from future contact with the adult justice system.

### PCL: YV Measured Juvenile Psychopathy: Risk Relevance and Prediction of Recidivism

The sample by and large tended to be lower in psychopathy than sexual offending youth samples elsewhere (Cale et al., 2015; Gretton et al., 2001; Parks & Bard, 2006; Viljoen et al., 2009), although the results underscored the forensic and criminal justice relevance of the juvenile psychopathy construct and its impact on others. Specifically, youth with prominent psychopathic features were more likely to score higher on a measure of actuarial sexual violence risk, and its risk-need domains (hence, having a greater number of criminogenic needs), and they were more likely to reoffend violently and generally (hence, being de facto, higher risk).

The largest convergent associations were observed between the PCL: YV lifestyle and antisocial facets with the VRS-YSO’s antisocial tendencies factor, indicating measurement of a common underlying construct of general antisociality that is correlated with risk. The PCL: YV’s interpersonal facet was also significantly correlated with the sexual deviance domain from the VRS-YSO and was the only component of the PCL: YV to evince such an association. Such a finding may suggest that interpersonal features such as manipulation, lying, and impression management are key factors for youth to gain access to victims and maintain a sexually deviant lifestyle. Finally, the broadly moderate significant associations between the PCL: YV’s lifestyle and antisocial facets and the VRS-YSO’s family concerns factor may reflect behavioral issues that are concordant with family stress and instability in a reciprocal manner.

PCL: YV scores, consistent with the extant literature on youth sexual offending samples as well as youth justice samples in general, predicted nonsexual violence, general violence, and any recidivism with moderate to high accuracy. This was particularly the case for the lifestyle and antisocial facets, although the affective facet also predicted general recidivism. The PCL: YV did not significantly predict sexual recidivism, although the effect sizes were in the small to moderate range and on par with the magnitude of effects in the adult literature (Hawes et al., 2013). PCL: YV total scores also did not significantly increment predictions of sexual, violent, or general recidivism beyond a purpose-built youth sexual violence risk measure. The weaker association with respect to sexual recidivism is to be expected given that that the PCL: YV is not a risk tool and does not measure risk constructs specific to sexual offending. For the violent and general recidivism incremental validity analyses, the PCL: YV and VRS-YSO had shared risk variance, and each generated broadly the same hazard ratio magnitudes for these outcomes when examined in the same regression block; as such, it is anticipated that the smaller sample size decreased power for these analyses.

### Juvenile Psychopathy and Treatment Response: Evidence for Risk Mitigation and Reduced Reoffending?

Rates of sexual recidivism remained low in the sample overall (8%), consistent with reports from the extant literature on court adjudicated samples (Caldwell, 2011; 2016), and nonsignificantly higher among youth scoring above average on the PCL: YV; yet nearly half (48%, or 10/21) of the youth scoring 20 or higher on the PCL: YV were eventually convicted for a new general violent offense and over 60% had any new offense. Although youth scoring in this range made up only about 20% of the sample, they represented about 40% of the youth who went on to commit new violent offenses and a similar proportion (35%) of youth with any future offense. To what extent was risk mitigated, and was potential harm and negative impacts prevented, through the provision of services had none been available?

Youth who were above average in psychopathy relative to the other youth in the sample were significantly less likely to complete full services through the adolescent sexual offense outpatient program. The lifestyle and interpersonal features had the strongest unique associations with noncompletion—what may explain this? The lifestyle features which reflect characteristics such as proneness to boredom, lack of goals, impulsivity, and irresponsibility that are extreme and atypical even for a teenager, could reflect poor therapeutic work ethic, lack of motivation, and unreliability with services. Youth with a number of these issues may be late for group, miss sessions, do not contribute in groupwork, are distractible and disruptive, or fail to complete necessary therapeutic tasks. The results are consistent with Smallbone et al. (2009) who found the impulsivity/antisociality subscale from the Juvenile Sex Offender Protocol-II (Prentky & Righthand, 2003) to correlate inversely with therapeutic engagement in an Australian sample of youth convicted for sexual offenses, as well as general findings from the adult sexual offending and psychopathy treatment literature (DeSorcy et al., 2020; Sewall & Olver, 2019). Further, youth with the interpersonal features may be deceitful and manipulative, display aggressive bravado, or by contrast heightened impression management and insincerity. The consequence of each of these sets of psychopathy features is the potential for frustration and challenges that strains the countertransference of service providers, as well creating the potential to undermine the progress of other youth. Regardless, as treatment interfering behaviors, they are issues to be managed to retain the youth in services for potential benefit, per the responsivity principle.

Importantly, PCL: YV score was not significantly associated with changes in risk on the VRS-YSO dynamic items, rated pre and posttreatment. That is, youth were registered as having made risk reduction irrespective of their levels of psychopathy. One possible exception was a mild positive association between interpersonal facet scores and change that remained after controlling for the other facets; antisocial facet scores were also nonsignificantly associated with decreased change. It is possible that the impression management and verbal tendencies captured by the interpersonal facet could have manifested in verbal cooperativeness and behavioral compliance in services, which were reflected in the change ratings, while higher levels of baseline antisociality could have slightly attenuated change and response to services. It is unlikely that the change scores solely reflected impression management, however, or were less risk relevant for youth with higher PCL: YV scores; after imposing stringent controls for baseline risk and individual differences in psychopathy, change scores were inversely associated with recidivism. Although the covariates in the final regression model (PCL: YV and change score) were associated at *p* < .10 with any recidivism, the analyses were based on a subset of 53 youth who had pre-post change information available and the magnitude of the hazard ratios were consistent with those found in the adult literature examining psychopathy and change associations with recidivism (see Olver et al., 2013; Sewall & Olver, 2019). Given that PCL: YV group ratings and treatment completion status had little association with general violent or any recidivism, while change scores had more promising associations with outcome, the findings suggest that progress in treatment probably matters more than simply completing it.

### Implications for Clinical Forensic Practice

The present study results have several implications for the use of the PCL: YV and the utility of the juvenile psychopathy construct for youth who have sexually offended in clinical-forensic contexts. First, the results support the use of the PCL: YV as a forensic measure to complement existing measures of risk and need in youth sexual violence risk assessment. The results support use of a specialized sexual offense measure to assess risk for future sexual offending, identify treatment targets, and to assess changes in risk. The PCL: YV would seem to provide additional information with respect to propensity for future violence or any reoffending—where it predicted strongest—and to identify interpersonal and emotional features to be managed in treatment and supervision. It is important to underscore responsible use of the tool in this context and the PCL: YV should not be used to label youths as psychopathic or not treatable (Olver & Stockdale, 2010). Rather, given that youth scoring high on the PCL: YV are also higher risk for general reoffending outcomes and have a greater volume of criminogenic needs, the higher scoring a youth happens to be, the more they should be prioritized for intervention and risk management services per the risk and need principles.

Second, the assessment of psychopathy-related personality features can assist with treatment planning by providing treatment providers information on certain characteristics (e.g., impression management, manipulativeness, and lack of prosocial emotions) that may impede treatment engagement and successful completion, if they are not managed by service providers, per the responsivity principle (Andrews et al., 2006). The present study results demonstrate that youth with above average traits of psychopathy and a history of sexual offending can be retained in services, make treatment relevant changes, and do not inexorably reoffend violently or otherwise. This is particularly important given that the majority of index sexual offense victims were family members, as well as other children. Reducing victimization in this sense is not only beneficial in mitigating the impacts of juvenile psychopathy on society in general, but also families and children, in particular, including the promotion of healthy families and community wellbeing.

Finally, an understanding of the ongoing developmental changes occurring throughout adolescence is essential to appreciate the potentially dynamic nature of psychopathy-related features, as measured by the PCL: YV. That is, the presence and/or degree of certain interpersonal, emotional, and behavioral features may vary at different points in time as a function of development; therefore, reassessments of these features would be informative. Other researchers have also found that scores on the PCL: YV predict short-term recidivism better compared to long-term (adult) recidivism among justice involved youth (e.g., Gretton et al., 2004; Stockdale et al., 2010). This finding along with an understanding of the dynamism of psychopathy-related features among certain justice involved youth serve to caution against making long-term decisions about a youth’s risk for recidivism and amenability for treatment (Viljoen et al., 2010).

### Limitations and Future Directions

The present study has limitations to be considered for the interpretation and contextualization of findings. First, as mentioned before, CPIC criminal records, albeit thorough as these capture youth and adult charges, convictions, and sentences, are an imperfect means for obtaining an accurate representation of criminal behavior as it captures only detected cases. Researchers elsewhere have recommended the use of additional sources to capture offending behavior such as self-report and treatment records (e.g., Viljoen et al., 2009). Moreover, given the study’s focus on community recidivism, and that the vast majority of treated youth were serving community sentences, we did not have information pertaining to other important outcome criteria, such as institutional sexual offending. Second, the study measures, and the PCL: YV specifically, was coded solely from youth files, which might have reduced the potential to fully capture traits in the interpersonal and/or affective facets of the PCL: YV. Future research should attempt to code the PCL: YV based on a combination of file review and clinical interviews, including collateral sources (e.g., family members).

A third limitation was the small sample size, particularly for treatment change and completion analyses with recidivism outcomes. The study jurisdiction is a geographically vast but sparsely populated region, such that several years of consecutive referrals for sexual offense specific services with sufficiently detailed files for clinical research purposes, still amounted to a relatively small sample. A larger sample might allow statistically significant differences or effects to be detected (e.g., psychopathy-related personality features, treatment status, and criminal outcome), particularly when examining associations with low base rate outcomes such as sexual recidivism. That the effect size magnitudes were consistent with prior psychopathy and sexual offense treatment change research (Olver et al., 2013; Sewall & Olver, 2019) may lend some credence to the potential stability of these findings.

Fourth, the present study examined the forensic and therapeutic role and relevance of the juvenile psychopathy construct on the criminal and treatment outcomes for the whole sample. Future research should attempt to examine various moderator variables such as age (e.g., Viljoen et al., 2009), ethnocultural diversity (e.g., Stockdale et al., 2010), and youth offending type (e.g., Parks & Bard, 2006) to better understand the nature and mechanisms of juvenile psychopathy in the offense dynamics and community outcomes of sexually offending youth. It would also be worthwhile to examine the predictive ability of the PCL: YV for youth and adult recidivism outcomes separately (see Stockdale et al., 2010). Finally, it would be useful to code the PCL: YV prior to and following treatment to assess change in psychopathy-related features and examine the association between these changes and recidivism outcome.

Notwithstanding these limitations, the present study contributes to a limited body of research examining the role of PCL: YV-measured psychopathy in the prediction of criminal and treatment outcomes among sexually offending youth. Adolescence is a period of transition and change, and youth with elevated psychopathy features can change for the better to improve wellbeing and reduce the potential for further victimization and harm within families and communities.
